# Initial Low Levels of Suicidal Ideation Still Improve After Cognitive Behavioral Therapy for Insomnia in Regular Psychiatric Care

**DOI:** 10.3389/fpsyt.2021.676962

**Published:** 2021-06-28

**Authors:** Susanna Jernelöv, Erik Forsell, Viktor Kaldo, Kerstin Blom

**Affiliations:** ^1^Centre for Psychiatry Research, Department of Clinical Neuroscience, Karolinska Institutet, Stockholm Health Care Services, Stockholm, Sweden; ^2^Division of Psychology, Department of Clinical Neuroscience, Karolinska Institutet, Stockholm, Sweden; ^3^Department of Psychology, Faculty of Health and Life Sciences, Linnaeus University, Växjö, Sweden

**Keywords:** suicidal ideation, cognitive behavioral treatment for insomnia, depression, prediction, insomnia

## Abstract

Insomnia disorder is highly prevalent, and has been identified as a risk factor for many psychiatric problems, including depression, suicide ideation and suicide death. Previous studies have found that cognitive behavioral therapy for insomnia (CBT-I) reduce depression and suicidal ideation in samples with high levels of suicidal ideation. This study aims to investigate associations of CBT-I with suicidal ideation in a sample of 522 patients primarily seeking internet-delivered treatment for insomnia in regular psychiatric care. The sample had high pretreatment insomnia severity levels and a relatively high level of comorbid depression symptoms. Suicidal ideation levels were relatively low pretreatment but still improved significantly after CBT-I. Contrary to previous findings, the strongest predictor of changes in suicidal ideation were improvements in depressive symptoms, rather than improvements in insomnia. We conclude that suicidal ideation may not be a major problem in these patients primarily seeking treatment for insomnia, despite comorbid depressive symptoms, but that suicidal ideation still improves following CBT-i. Considering the increased risk for patients with untreated insomnia to develop depression, this finding is of interest for prevention of suicidal ideation.

## Introduction

Insomnia is a very common problem, with around 11% of the general population fulfilling criteria for a diagnosis of insomnia disorder (1, 2). Insomnia disorder is often comorbid with other psychiatric problems such as depression, and has been identified as a risk factor for depression (3, 4), suicidal ideation (5) and even suicide death (6). Associations between insomnia and suicide have been found both in patients with increased suicide risk, e.g., depressed patients and veterans, but also in community samples (7). Some studies have shown that changes in insomnia symptoms predict changes in suicidal ideation (8). Consequently, it has been suggested that treating insomnia may play a role in preventing both depression (9) and suicide (10).

Indeed, it has been shown that cognitive behavioral therapy for insomnia (CBT-I) may alleviate depression (11), also for those patients suffering from both diagnoses (12). A limited number of recent studies have shown that suicidal ideations can improve with treatment of insomnia with CBT-I in veterans (13, 14). Treating insomnia with hypnotics has also been tested in patients with major depressive disorder, insomnia and suicidal ideation (15), and although treatment did not directly improve suicidal ideation when comparing Zolpidem to placebo, reduction in suicidal ideation was correlated with improvement in insomnia. The studies by Trockel et al. (14) and McCall et al. (15) both find that improvements in insomnia, rather than improvements in depressive symptoms, drive the reductions in suicidal ideation (14, 15).

These findings are important, and provide some support for the use of interventions to treat insomnia as a means to reduce the risk of suicide in patients with increased risk. However, it is possible that associations between insomnia, depressive symptoms and suicidal ideation may not be uniform across all patient groups (7), and so the findings may not be directly generalizable to a general psychiatric sample with insomnia.

The present study therefore aimed at investigating the prevalence of depression and suicidal ideation in consecutive patients with insomnia being treated for their insomnia at the Internet Psychiatry Clinic, Stockholm County Council; whether depressive symptoms and suicidal ideation improved during treatment; and the nature of the associations between changes in suicidal ideation, and symptoms of depression and insomnia during internet-delivered CBT-I.

## Materials and Methods

### Procedure and Setting

The participants in this study were patients at the Internet Psychiatry Clinic within the specialized psychiatry services, being a part of the public health services in Sweden (16). The clinic operates like a conventional outpatient clinic, although also allowing for self-referrals, and provides face-to-face assessment performed by physicians and assumes full responsibility for the patient while in their care. The Internet Psychiatry Clinic offers treatment for depression, several anxiety disorders, irritable bowel syndrome and insomnia. More than 6,000 patients have, to date, received treatment with therapist-guided internet-delivered cognitive behavioral therapy (ICBT). Other health care providers can refer patients, but the vast majority of patients (~98%) apply for treatment through a secure, government managed website. When applying, patients fill out a number of questionnaires and symptom scales, and indicate which treatment they believe would best fit their needs. All patients are then called to a face-to-face assessment meeting with a psychiatrist, or a physician under psychiatrist supervision. The assessment includes a structured anamnestic interview, a diagnostic interview (M.I.N.I.), with an addition for diagnosing Insomnia, and a symptom severity assessment. Patients who are not included for treatment at the Internet Psychiatry Clinic but require other treatment are referred within the health care services.

This study was approved by the Regional Ethics Board in Stockholm, Sweden (2018/2550-32).

### Participants

Inclusion criteria were: 16 years or older; diagnosed with insomnia disorder according to the DSM-5 criteria; not primarily being in need of another assessment or treatment; stable (since 4–8 weeks) or no use of antidepressant medication; not having a bipolar disorder or a psychotic disorder, or being severely depressed or suicidal according to the assessor; no alcohol or drug dependency that is likely to interfere with treatment; able to read and write in Swedish. Sleep medication use or other comorbid disorders are not grounds for exclusion.

In the clinic, of patients seeking treatment for insomnia, approximately 80% are included for treatment following the assessment process. Of those not included, about one-third need further or other assessments (e.g., other sleep disorders, neuropsychiatric disorders, bipolar disorder or psychosis), about one-third turn down treatment after assessment, and the last one-third are excluded for a large number of other, rare, reasons. For instance, <0.5% are excluded due to high levels of suicidal ideation, and another 0.5% due to their depression being too severe. Of the patients who are included for treatment, the vast majority (about 98%) receive the treatment they initially indicated would best fit their needs. The patients do not simultaneously receive ICBT for another condition.

### Treatment

The insomnia treatment used in this study is a 9-week therapist-guided internet-delivered cognitive behavioral therapy for insomnia (ICBT-i). The treatment was implemented in September 2017, following randomized controlled trials: the first study comparing ICBT-i to an active control treatment (17, 18) and the first study comparing ICBT-i to a face-to-face treatment (19), showing that the treatment was non-inferior to group treatment and had large within-group effect sizes.

The treatment is divided into eleven so called modules, or chapters, consisting of text to read, exercises and home-work assignments (see Table 1 for treatment content). Patients are initially given access to modules 1–3, and after sending in home-work assignments and receiving therapist feed-back for these three modules, are given access to modules 4–5. Thereafter, the patient gets access to one module at a time after receiving feed-back from the therapist.

**Table 1:** ICBT-i treatment content.

**Module**	**Title**	**Content**
1	Introduction	How the platform works, facts about sleep. What is sleep, what is it for? How much sleep do we need, and what are consequences of sleeploss? Is there such a thing as morning and evening people? What is insomnia?
2	The CBT-model	More about insomnia—how do we get insomnia and why does it not remit spontaneously? Introduction to cognitive behavioral therapy. Information about sleep medication and creating a cessation plan (optional).
3	Tired or sleepy? Myths about sleep	Learning the difference between feeling tired and being sleepy. Learning about the myths about sleep that may exacerbate insomnia.
4	Sleep rhythm	Introduction to relaxation and visualization exercises, and sleep restriction. Sleep restriction is the main intervention and is followed-up throughout the treatment.
5	Bed-signals	Introduction to stimulus control and getting started. Stimulus control is followed-up throughout the treatment.
6	Daytime and bed-time	Routines for getting daylight and exercise during the day and creating bed-time routines for consistency and winding down before going to bed
7	This is where you are	Acceptance, mindfulness, attitudes and expectations about sleep
8	Dealing with your thoughts	Cognitive reappraisal and behavioral experiments focused on thoughts, rumination and worry about sleep
9	Sleep Hygiene - keep your sleep clean	Information and exercises about sleep hygiene such as avoiding caffeine later in the day, not drinking alcohol close to bedtime, keeping your bedroom cool and dark, and not having heavy meals at night
10	More thoughts, more acceptance	Additional materials for working more with mindfulness, acceptance and cognitive reappraisal
11	Progress so far and moving forwards	Summary, plan for the future and relapse prevention

The therapists at the Internet Psychiatry Clinic are licensed clinical psychologists with thorough training in CBT. Patients read educational texts, do homework assignments, fill out work-sheets and a sleep diary, all on-line. Therapists and patients communicate within the secure treatment platform using an email-like service, where therapists give feed-back and support regarding the work the patient does, and patients can ask questions. The written contacts occur at least once per week and up to three times per week. Therapists respond to patient homework or questions within 48 h. If required, telephone calls can be used to sort out complicated issues, but this is very rare and for most patients, telephone contact does not occur during treatment.

Measurements of insomnia and depressive symptoms are administered before, during and after the 9-week long treatment, in order to monitor progress, sudden deterioration, and changes in suicidal ideation. If a patient indicates increased levels of suicidal ideation (above four points on MADRS-S item 9, see below), a structured telephone interview is conducted and, if necessary, the patient comes to the clinic for a face-to-face assessment.

### Measures

**Background data** on age, gender, educational level, household status, sick-leave and employment status were collected via screening questionnaires and intake interviews. To facilitate comparison with other studies, baseline score for the Patient Health Questionnaire [PHQ-9; total score 0–27; (20)] is included but is not used as an outcome.

**Adequate treatment dose** was defined as having access to 5 out of 11 treatment modules. Sleep restriction is introduced in module 4 and stimulus control in module 5 (see Table 1 for details on treatment content). The intention is that the first five modules should be completed within the first two weeks of treatment, although many patients progress slower through the treatment.

**Insomnia severity** was measured with the Insomnia Severity Index (ISI), (21). The scale has shown good psychometric properties and is the most commonly used insomnia scale. ISI has seven items scored from 0–4, giving a total score ranging from 0–28 with higher scores indicating more severe insomnia.

**Depression severity** was measured with The Montgomery Åsberg Depression Rating Scale–Self rated (MADRS-S), (22). It has nine items scored from 0–6 points, with text descriptions provided for 0, 2, 4, and 6 points, and has adequate psychometric properties. The total score ranges from 0–54 with higher scores indicating more severe depression. The much-used 12- point cut-off was used for detecting (mild) depression (23).

**Modified depression**. In order to explore relations between depression, insomnia, and suicidal ideation in a methodologically more unbiased way, a modified measure of depression was constructed from the MADRS-S total score, but without item 9 (suicidal ideation) and item 3 (sleep problems) and included as a control variable in the prediction model.

**Suicidal ideation** was measured using MADRS-S item 9. The options when scoring are (our translation from Swedish):

0 p: I have a normal appetite for life
1p (Textless)
2 p: Life does not seem meaningful but I still do not wish I was dead
3p (Textless)
4 p: I often think it would be better to be dead, and although I do not wish for it, suicide can sometimes feel like an option/a way out
5p (Textless)
6 p: I am convinced that my only way out is to die, and I think a lot about how to best go about taking my life.

### Statistics

Means and standard deviations are reported for observed values, and used to calculate Cohen's *d* with the formula Mean(pre)-Mean(post)/Pooled Standard Deviation (i.e., positive values indicate a decrease from pre to post).

Linear mixed models were used to analyze change (slope) in depression and suicidal ideation from pre- to post-treatment. Each model included nine time-points i.e., pre-treatment, seven weekly measures, and post-treatment. To improve model fit, intercept and time were included as random effects and a first order autoregressive covariance structure was employed.

For the prediction analyses, we used hierarchical linear regression to predict pre to post change in the suicide item, introducing sleep variables in step one, depression variables in step two, and finally age, sex, and education as control variables into the model in step three. Primary analyses were made with the whole sample according to intent-to-treat, using the last available measurement for all patients who provided at least one assessment point after the pre-treatment assessment. We then performed three sensitivity analyses using the same linear regression model used in the primary analysis: (i) for completers, i.e., those who had received an adequate treatment dose (5/11 modules), (ii) only for those who had a depression severity above 12 points on MADRS-S at the pretreatment assessment, and (iii) only for those who scored two or more points on the suicide item at baseline.

Missing data was handled through the use of linear mixed models, and through the use of “last available measure” in the linear regression model.

SPSS 27 was used for the analyses (IBM Corp. Released 2020. IBM SPSS Statistics for Windows, Version 27.0. Armonk, NY: IBM Corp).

## Results

### Attrition

Of the 552 participants, post-treatment assessments were completed by 445 (81%), and at least one measure after baseline was obtained from 537 patients (97%).

### Participants

We included all patients who received ICBT-i at the clinic from the introduction of the treatment in September 2017 to November 2020, N=552. For baseline patient characteristics, see Table 2.

**Table 2:** Demographics and pretreatment symptom levels for all participants (*n* = 552).

**Variable**	**Descriptives**
Age, Mean (SD) Range	44 (13) 16–83
Female, *n* (%)	361 (66%)
Single household, *n* (%)	154 (28%)
Tertiary education, *n* (%)	379 (69%)
Employed or student, *n* (%)	477 (86%)
On 50% sick leave or more, *n* (%)	13 (2%)
Years with insomnia, *n* (%)	
<1 year	38 (7%)
1–5 years	138 (25%)
6–10 years	69 (13%)
More than 10 years	272 (50%)
Missing data	35 (5%)
MADRS-S, Mean (SD)	14.6 (7.1)
Modified depression (i.e., MADRS-S without sleep and suicide-items), Mean (SD)	10.2 (6.0)
Depression diagnosis (estimated with MADRS-S > 12), *n* (%)	317 (57%)
PHQ-9, Mean (SD)	7.9 (4.2)
ISI, Mean (SD)	20.3 (4.0)

*MADRS-S, Montgomery Åsberg Depression Rating Scale–Self rated; PHQ-9, Patient Health Questionnaire 9 items; ISI, Insomnia Severity Index; SD, standard deviation*.

### Treatment Adherence

Two hundred and forty seven patients (44.7%) finished all treatment modules. One hundred four patients (19%) did not finish the first five modules, i.e., did not receive “an adequate dose” of treatment. On average patients received 8.7 of the 11 modules.

### Prevalence of Depression and Suicidal Ideation

As can be seen in Table 2, more than half of the patients scored above cut-off for mild depression. Table 3 displays the prevalence of different levels of suicidal ideation, with 81% of participants endorsing normal or close to normal appetite for life (56 and 25%, respectively), and only 1% endorsing seeing suicide as a solution to their problems.

**Table 3:** Frequencies of scores for the MADRS-S suicidal ideation item.

**MADRS-S suicidal ideation item 9 frequency**	**Pre-treatment assessment *n* = 552**	**Last available measure^a^*n* = 537**
Normal appetite for life 0	309 (56%)	324 (60%)
1	136 (25%)	123 (23%)
Not so meaningful, but no wish to die 2	86 (15%)	75 (14%)
3	17 (3%)	13 (2.4%)
Often think better off dead, suicide can be a way out 4	4 (1%)	1 (0.2%)
5	0 (0%)	0 (0%)
Convinced death the only way, think a lot about how to die 6	0 (0%)	1 (0.2%)

a *Posttreatment for 445 participants and last available weekly measure for 92 participants; MADRS-S, Montgomery Åsberg Depression Rating Scale–Self rated*.

### Changes in Depressive Symptoms and Suicidal Ideation During Insomnia Treatment

Symptoms of depression, i.e., total score on MADRS-S, improved from *M* = 14.6 (SD = 7.1) at pre-treatment to *M* = 9.3 (SD = 7.6) at post-treatment (observed data, Cohen's *d* = 0.72 [CI95% 0.59–0.85]). The slope of the linear mixed models analysis was statistically significant (*t* = −15.7, df = 534.6, *p* < 0.001), and the estimated mean post value was 9.5 (SE = 0.31).

Suicidal ideation also improved from *M* = 0.68 (SD = 0.90) at pre-treatment to *M* = 0.54 (SD = 0.82) at post-treatment (observed data, Cohen's *d* = 0.16 [CI95% 0.04–0.29]). The slope of the linear mixed models analysis was statistically significant (*t* = −3.35, df = 529.2, *p* = 0.001), and the estimated mean post value was 0.56 (SE = 0.035). See Table 3 for the frequencies of scores at pre- and post-treatment assessments.

### Prediction of Change in Suicidal Ideation

The primary predictive analysis was made with the last available measure for all patients with at least one completed measurement point after baseline (*n* = 536). As can be seen in Table 4, although change in insomnia severity predicted change in suicide ideation in the first step, this association was no longer statistically significant when baseline depression symptoms and change in depression symptoms was included in step two. The covariates in step three were not statistically significant and did not change the results.

**Table 4:** Prediction of decrease in suicidal ideation from pre- to post-treatment.

**Model/Step number (Explained variance)**	**Unstandardized coefficients**		**Standardized coefficient**		**95% CI for B**	
**Variable**	**B**	**Std. err**.	**β**	***p***	**Lower**	**Upper**
**Step 1**(r^2^ = 0.33; *p* < 0.001)						
Initial suicidal ideation	0.38	0.028	0.51	<0.001	0.33	0.44
Initial insomnia severity	−0.04	0.007	−0.20	<0.001	−0.05	−0.02
Pre-post change insomnia severity	0.05	0.005	0.42	<0.001	0.04	0.06
**Step 2**(Δr^2^ = 0.21; *p* <0.001)						
Initial suicidal ideation	0.52	0.030	0.69	<0.001	0.46	0.58
Initial insomnia severity	0.002	0.006	0.01	0.79	−0.01	0.01
Pre-post change insomnia severity	0.004	0.005	0.03	0.46	−0.01	0.01
Initial depression (modified)	−0.06	0.005	−0.48	<0.001	−0.07	−0.04
Pre-post change depression (modified)	0.08	0.005	0.62	<0.001	0.07	0.09
**Step 3**(Δr^2^ = 0.01; *p* = 0.103)						
Initial suicidal ideation	0.53	0.030	0.69	<0.001	0.47	0.58
Initial insomnia severity	0.002	0.006	0.01	0.78	−0.01	0.01
Pre-post change insomnia severity	0.003	0.005	0.03	0.51	−0.01	0.01
Initial depression (modified)	−0.06	0.005	−0.50	<0.001	−0.07	−0.05
Pre-post change depression (modified)	0.08	0.005	0.62	<0.001	0.07	0.09
Age	−0.002	0.002	−0.05	0.12	−0.005	0.001
Gender	0.08	0.042	0.06	0.054	−0.001	0.17
Education	0.03	0.044	0.02	0.57	−0.06	0.11

*Suicidal ideation, Item 9 in The Montgomery Åsberg Depression Rating Scale–Self rated (MADRS-S); Insomnia severity, Insomnia Severity Index (ISI); Depression (modified), MADRS-S sum, excluding item 9 on suicidal ideation and item 3 on sleep problems; For gender, 1 = male and 2 = Female; For education, 0 = no tertiary education and 1 = tertiary education*.

Three sensitivity analyses were performed, all also using the last available measure after baseline. The association between change in insomnia and change in suicidal ideation in step 2, i.e., when controlling for initial depression and change in depression, remained non-significant through all sensitivity analyses. However, the estimated B grew slightly from its level of 0.004 in the primary analysis, when the analysis was made on the 485 completers (*B* = 0.005), on the 306 patients with with initial depression severity indicating probable diagnosis (*B* = 0.010), and on the 106 patients that initially scored 2 or more points on the suicide item before treatment (*B* = 0.022). In the latter sub-group, *p* was 0.08 and thus rather close to being statistically significant.

## Discussion

In this study, we aimed at investigating the prevalence of depression and suicidal ideation in consecutive patients with insomnia receiving internet-delivered CBT-I within psychiatric care, whether depressive symptoms and suicidal ideation improved with treatment, and the nature of the associations between changes in suicidal ideation, symptoms of depression, and symptoms of insomnia during internet-delivered CBT-I.

At baseline, 57% of patients were estimated to have a depression diagnosis and the pre-treatment level of depressive symptoms was, on average, in the mild to moderate range. Furthermore, depressive symptoms improved during treatment. This is not a new finding; the relationship between insomnia treatment and improvements in depression, and even prevention of depression, has been established before (9, 11, 12).

However, depressive symptoms are not synonymous with suicidal ideation, and although most scales measuring depressive symptoms include items for suicidal ideation, this construct needs to be analyzed separately.

In this sample, the majority of participants indicated no suicidal ideation at pre-treatment. Nevertheless, despite this floor-effect and in line with previous research in depressed patients and veterans (13–15), suicidal ideation showed an overall improvement among insomnia patients receiving CBT-i. Interestingly, contrary to some previous studies (14, 15), in the present study improvements in depressive symptoms, when excluding items for sleep problems and suicidal ideation, was the strongest predictor of reductions in suicidal ideation, not improvements in insomnia severity. This was true also in our sensitivity analyses with patients completing the most important part of the CBT-I program and with patients estimated to have a depression diagnosis at baseline. It was also true for the smaller sub-group of patients with two or more points on the suicidal ideation item, although in this analysis the contribution of improvements in insomnia severity was close to significant.

There may be several reasons for the difference between our results and previous studies. First, the current study used a markedly different sample compared with studies that had suggested improvements in insomnia, rather than improvements in depressive symptoms, as driving the reductions in suicidal ideation. Trockel et al. (14) and Bishop et al. (10) both studied U.S. veterans. Rates of suicide in U.S. military veterans are high and exceed the civilian population by 40–60% (24), making those samples highly specific and the findings hard to generalize. McCall et al. (15) on the other hand, specifically selected patients with suicidal ideation, i.e., their sample represents a subpopulation of people suffering from insomnia, who also have suicidal ideation.

Our findings show that significant suicidal ideation was not highly prevalent in these patients primarily seeking help for their insomnia. Our sample was rather different from the previous studies, considering they did not present initial high levels of depressive symptoms or suicidal ideation, and possibly had a higher general level of functionality since they had self-referred to internet-delivered CBT-I and showed a high educational level and were mostly at work. Our sub-sample with higher initial suicidal ideation mimicked previous results somewhat more, although reductions in insomnia in this sub-sample were still not statistically significantly related to reductions in suicidal ideation, when controlling for depression. It is possible that the internet-delivered format is more suitable for patients with a higher level of functioning, and that the level of functioning is something that also affects levels of suicidal ideation, and the nature of the relationship between depression, insomnia and suicidal ideation. These hypotheses would need to be tested in further studies.

Second, it has been consistently shown that suicidal ideation is related to insomnia. It has also been shown that in individuals with suicidal ideation and sleep problems, treatment of insomnia may provide relief and reduce risk. While it is most important that improved sleep is a viable way to reduce suicidal ideation in such samples, this does not equate to suicidal ideation being inherently strongly associated with sleep in a population otherwise relatively low in suicidal ideation. We were therefore interested to see if insomnia itself was related to suicidal ideation, i.e., if the patients who came to our clinic seeking help primarily for their insomnia also had high levels of suicidal ideation. This was not the case. On the contrary, our study shows that levels of suicidal ideation are low in this help-seeking sample of Swedish adults with insomnia. Whether this is true also in other samples of patients seeking help primarily for insomnia, is an empirical question that would merit further investigation.

Third, the fact that the association between reductions in insomnia and suicidal ideation found in the first step in our regression model disappeared in the second step, when change in depression was added, does not say anything about causal relations. Since the data collection was performed during an intervention that has been shown to cause reductions in insomnia severity, it is not very far-fetched to assume that the reduction in depression, which the model suggests was leading to lower suicidal ideation, was itself caused by a reduction in insomnia severity. From this perspective, CBT-I could have caused the decrease in suicidal ideation, with levels of depression acting as a mediator. To look more closely at this hypothesis, future studies could perform mediational analyses according to this theoretical model.

### Limitations

The main limitation is the suicidal ideation-item in the MADRS-S scale itself. The responses for the lower scores do not actually refer to degrees of thinking of, or wanting to, die or commit suicide, but rather to a diminished zest for life or finding life meaningless but not wanting to die. Other scales for measuring suicidal ideation might better capture the degree by which the respondent is contemplating suicide, also when the intention is very low.

Another limitation is the use of self-report measures only. Although the scales used have adequate psychometric properties including high validity, the inherent problems with self-report assessments still need to be taken into consideration when interpreting the results. Another, related issue, is that all data coming from the same source—i.e., the patient—might inflate the relationship between the studied factors.

Another limitation is the low variance in responses to the suicidal ideation item in the sample and the fact that the distribution seems to be bounded by zero, which is hard to avoid due to the nature of the research question and operationalization of suicidal ideation being rather one-sided. This does affect the assumptions of some of the statistical analyses used, though the large sample should minimize the risk of spurious associations. Also, this problem is mitigated as the prediction analyses were carried out using change score for the suicidal ideation item, which did not suffer from a floor-effect.

## Conclusions

Suicidal ideation was not a common occurrence in a Swedish sample of insomnia patients primarily seeking internet-delivered CBT-I within regular psychiatry, even though more than half of the patients presented with elevated levels of depressive symptoms. Despite the initially low levels, suicidal ideation was further reduced following CBT-I. Contrary to previous findings in more suicidal and depressed samples, the reduction in suicidal ideation was more strongly associated with improvements in depression than with improvements in insomnia in this mostly self-referred and presumably rather well-functioning sample. Considering the increased risk for patients with untreated insomnia to develop depression, these findings are of interest for prevention of suicidal ideation, but need to be further investigated in future studies.

## Data Availability Statement

The datasets generated for this article are not readily available because Patient data sharing is restricted by Swedish law. Requests to access the datasets should be directed to susanna.jernelov@ki.se.

## Ethics Statement

The studies involving human participants were reviewed and approved by Swedish Ethical Review Authority (2018/2550-32). Written informed consent for participation was not required for this study in accordance with the national legislation and the institutional requirements.

## Author Contributions

All authors listed have made a substantial, direct and intellectual contribution to the work, have approved it for publication and agreed to be accountable for all aspects of the work.

## Conflict of Interest

The authors declare that the research was conducted in the absence of any commercial or financial relationships that could be construed as a potential conflict of interest.
